# Causal relationship between particulate matter 2.5 and hypothyroidism: A two-sample Mendelian randomization study

**DOI:** 10.3389/fpubh.2022.1000103

**Published:** 2022-11-25

**Authors:** Yuning Zhang, Shouzheng Liu, Yunwen Wang, Yue Wang

**Affiliations:** ^1^College of Environment, Liaoning University, Shenyang, Liaoning, China; ^2^Liaoning Provincial Ecological and Environmental Affairs Service Center, Shenyang, Liaoning, China; ^3^National Center for Human Genetic Resources, Beijing, China; ^4^Department of Environmental Health, School of Public Health, Key Laboratory of Environmental Health Damage Research and Assessment, China Medical University, Shenyang, Liaoning, China

**Keywords:** PM_2.5_, hypothyroidism, Mendelian randomization, air pollution, GWAS

## Abstract

**Background:**

Epidemiological surveys have found that particulate matter 2.5 (PM_2.5_) plays an important role in hypothyroidism. However, due to the methodological limitations of traditional observational studies, it is difficult to make causal inferences. In the present study, we assessed the causal association between PM_2.5_ concentrations and risk of hypothyroidism using two-sample Mendelian randomization (TSMR).

**Methods:**

We performed TSMR by using aggregated data from genome-wide association studies (GWAS) on the IEU Open GWAS database. We identified seven single nucleotide polymorphisms (SNPs) associated with PM_2.5_ concentrations as instrumental variables (IVs). We used inverse-variance weighting (IVW) as the main analytical method, and we selected MR-Egger, weighted median, simple model, and weighted model methods for quality control.

**Results:**

MR analysis showed that PM_2.5_ has a positive effect on the risk of hypothyroidism: An increase of 1 standard deviation (SD) in PM_2.5_ concentrations increases the risk of hypothyroidism by ~10.0% (odds ratio 1.10, 95% confidence interval 1.06–1.13, *P* = 2.93E-08, by IVW analysis); there was no heterogeneity or pleiotropy in the results.

**Conclusion:**

In conclusion, increased PM_2.5_ concentrations are associated with an increased risk of hypothyroidism. This study provides evidence of a causal relationship between PM_2.5_ and the risk of hypothyroidism, so air pollution control may have important implications for the prevention of hypothyroidism.

## Introduction

Hypothyroidism refers to thyroid hormone deficiency. The diagnosis is mainly based on serum thyroid-stimulating hormone (TSH) and free thyroxine (FT4) levels (1). As a common condition, the prevalence of clinical hypothyroidism is ~0.2–5.3% in the general European population and 0.3–3.7% in the United States (2). Hypothyroidism can lead to an increased risk of hyperlipidemia and the development of cardiovascular disease and even heart failure, somatic and neuromuscular symptoms, reproductive disorders, and other adverse outcomes (3). Due to the widespread use of thyroid function tests (4), researchers have focused on exploring the factors that contribute to hypothyroidism, and there is growing evidence of the detrimental effects of exposure to environmental factors on thyroid function (5–8).

Epidemiological studies have shown that exposure to nitrogen dioxide (NO_2_) and carbon monoxide (CO) is associated with increased TSH and decreased FT4 (9). Particulate matter (PM) significantly affects the binding of thyroxine to transthyretin and reduces thyroxine levels (10). In 2015, a survey of about 15.1 million neonates in China showed that maternal exposure to air pollution during pregnancy may affect fetal thyroid development (8). Moreover, the survey revealed that PM_2.5_ exposure levels are positively associated with the risk of congenital hypothyroidism in offspring (8). To date, evidence on whether air pollution exposure impairs thyroid function remains limited, and current observational studies cannot confirm a causal relationship between air pollution and hypothyroidism.

When assessing the health risks of environmental pollutant exposure, consideration of genetic polymorphisms may provide better insights into individual environmental health risks. Previous studies have shown that adverse health outcomes due to environmental exposures are influenced by changes in gene expression (11, 12). Women carrying the *GPX4*-rs376102 AC/CC genotype are more sensitive to air pollutants and more likely to have preterm births (13). At high exposure levels of PM_10_, ozone (O_3_) and mean pollution standard index (PSI), children carrying the thrombomodulin-33G/A polymorphism (GA + AA genotype) are at higher risk of atherosclerosis (14). However, no studies have examined the combined effect of genetic polymorphisms and PM_2.5_ on the risk of hypothyroidism. With the advent of the post-genome-wide association study (GWAS) era, many efforts have been made to move beyond genetic associations to causality and mechanistic examination. Mendelian randomization (MR) uses single nucleotide polymorphism (SNP) as an instrumental variable (IV) and integrating existing GWAS summary statistics for causal inference. Supported by the fact that parental alleles are randomly assigned at the time of conception, the MR design is similar to a randomized controlled trial, that can effectively avoid the influence of confounding factors and reverse causality (15). A large number of GWAS provide an abundant data resource for MR studies (16). Many scholars have used MR studies to explore the causal relationship between hypothyroidism and systemic lupus erythematosus, hepatocellular carcinoma, and type 1 diabetes (17–19). However, the causal relationship between PM_2.5_ and hypothyroidism remains unclear.

Taken together, we have raised the hypotheses that these PM_2.5_ exposure may causally contribute to the development of hypothyroidism. In order to address the important gap in literature regarding this research question, we performed a two-sample Mendelian randomization (TSMR) study using the GWAS dataset publicly available on the IEU Open GWAS database to evaluate the causal relationship between PM_2.5_ concentrations and the risk of hypothyroidism.

## Materials and methods

### Study design and data sources

We conducted a TSMR analysis base on the summary-level data from the IEU Open GWAS database (https://gwas.mrcieu.ac.uk/datasets). The exposure data were PM_2.5_ GWAS summary dataset. The outcome data were GWAS summary dataset. The personal data of the subjects in this study was obtained from the UK Biobank, a large prospective study with over 500,000 UK participants (20). The detailed procedures for phenotyping, genetic detail, genome-wide genotyping, imputation and quality control of UK Biobank participants have been described elsewhere (21, 22). All participants had given informed consent in the corresponding original studies.

The PM_2.5_ GWAS summary dataset (GWAS ID:ukb-b-10817) included 423,796 participants of European ancestry. PM_2.5_ concentrations at participants' home addresses were estimated using a Land Use Regression (LUR) model (23).

The hypothyroidism GWAS summary dataset (GWAS ID:ukb-b-19732) contained 462,933 individuals of European descent, including 22,687 cases and 440,246 controls. Hypothyroidism cases were defined on the basis of clinical diagnosis and self-reported. Supplementary Table 1 presented the demographics of the patients included in GWAS summary dataset.

### Selection of instrumental variables

**Figure 1:**
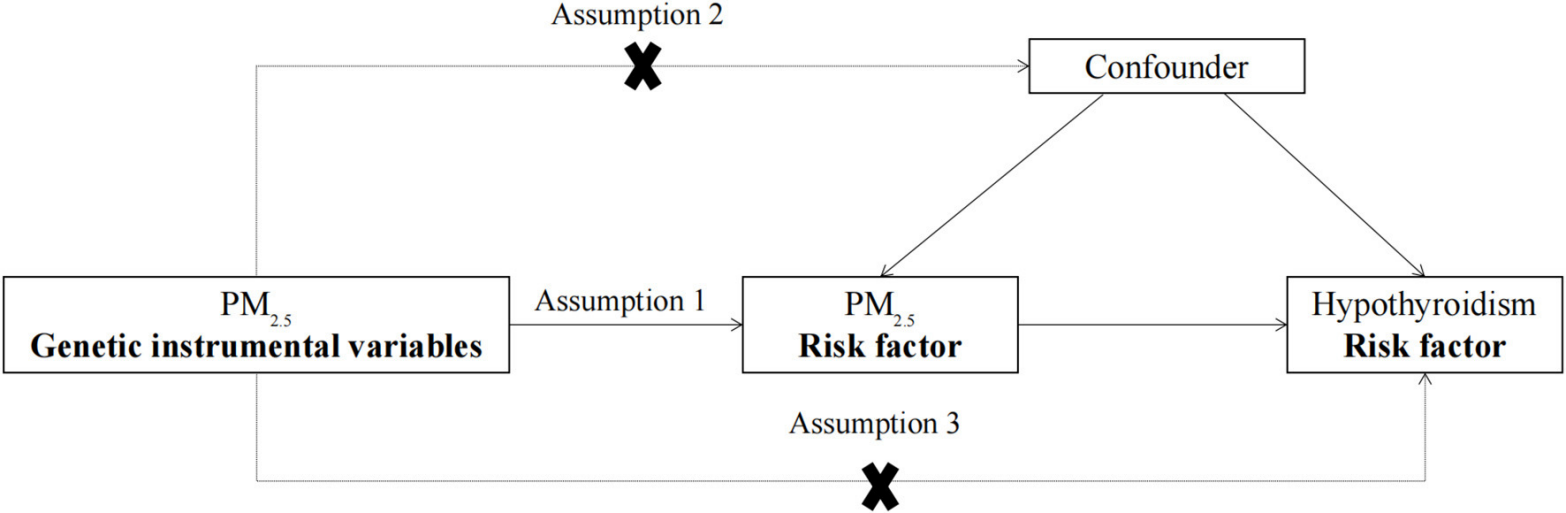
The design flow chart for the MR study. MR assumptions: assumption 1, 2, and 3. The solid line represents direct putative causal effects that PM_2.5_ genetic instrumental variants are reliably associated with I PM_2.5_ levels and influence the risk of hypothyroidism through the PM_2.5_ in assumption 1. The dotted line represents that PM_2.5_ genetic instrumental variants are not associated with any measured and unmeasured confounders and do not influence the risk of hypothyroidism through other pathways in assumptions 2 and 3, respectively. MR, Mendelian randomization; PM_2.5_, particulate matter 2.5.

As shown in Figure 1, to construct valid IVs, genetic variation must satisfy the three assumptions of MR. (1) Genetic IVs of PM_2.5_ are significantly associated with PM_2.5_ exposure levels. (2) The association between genetic IVs of PM_2.5_ and hypothyroidism is independent of confounding factors. (3) Genetic IVs of PM_2.5_ can only affect hypothyroidism risk through PM_2.5_ exposure. The study followed the Strengthening the Reporting of Observational Studies in Epidemiology using Mendelian Randomization (STROBE-MR) guideline (24), and the STORBE-MR checklist is provided in the Supplementary Table 2.

To meet assumption 1, we selected the corresponding single nucleotide polymorphisms (SNPs) for PM_2.5_ exposure at the threshold of genome-wide significance (*P* < 5 × 10^−8^). Linkage disequilibrium (LD) was estimated between SNPs to select independent genetic variants using clump parameter in R version 4.1.3 software (distance window 5,000 kb, linkage disequilibrium coefficient *r*^2^ <0.01 using the *R* packages “TwoSampleMR”) (25). We found eight SNPs that are significantly associated (*P* < 5 × 10^−8^) with PM_2.5_ exposure levels without LD, as shown in Supplementary Table 3.

In our MR analysis, pleiotropy testing was required to ensure that IVs did not influence hypothyroidism risk through other confounders or other biological pathways independent of PM_2.5_ exposure. The MR-Egger regression effects model can provide pleiotropy-corrected causal estimates in MR, assessing instrument strength independently of the null causality hypothesis under the direct effect assumption (26). Moreover, the method can work even if all selected SNPs are not unbiased estimates (26). By judging whether there is statistical significance between the intercept and 0, it indicates whether there is horizontal pleiotropy in IVs. MR-PRESSO enables a systematic assessment of the role of pleiotropy in MR (27). It includes three components: MR-PRESSO global testing, MR-PRESSO outlier testing, and MR-PRESSO distortion testing. The statistical threshold for IVs that may have horizontal pleiotropy is *P* < 0.05.

We used Cochran's Q test to evaluate heterogeneity in the estimates of heterogeneity calculated by the inverse-variance weighting (IVW) and MR-Egger models (28, 29). *P* > 0.05 indicated no significant heterogeneity in the screened IVs.

### TSMR analysis

In this TSMR study, we used IVW, MR-Egger, weighted median model, simple model, and weighted model methods to evaluate the causal relationship between PM_2.5_ exposure and hypothyroidism risk (30–32). The basic idea of IVW is to use the Wald ratio to obtain estimates of causal effects based on a single genetic IV, and then select a fixed-effects model to meta-aggregate multiple estimates of causal effects based on a single genetic IV. The IVW estimate is the combined causal effect estimate (33). IVW can provide reliable causal estimates without directed pleiotropy and is widely used in MR studies (34). We used MR-Egger, weighted median model, simple model, and weighted model methods to verify the causal association between exposure factors and outcomes. This endeavor has improved the accuracy of the findings (30).

### Sensitivity analysis

We used the leave-one-out method to analyze the influence of a single SNP on the results of TSMR analysis (35). By removing SNPs one by one and performing a meta-analysis on the remaining SNPs, we estimated the MR result including all remaining SNPs and compared it to the result with all SNPs. If the MR result changes significantly after excluding one SNP, then this SNP may be directly related to the results, violating assumption 3 (36).

### Statistical analysis

We conducted statistical analysis using *R* version 4.1.3 (R Foundation for Statistical Computing, Vienna, Austria) using the packages “TwoSampleMR” (25) and “MR-PRESSO” (27). The threshold of statistical significance for evidence of pleiotropy is *P* < 0.05.

## Results

### Extraction of genetic IVs of PM_2.5_ from the hypothyroidism GWAS dataset

We extracted corresponding information for seven genetic variants associated with PM_2.5_ concentrations in the hypothyroid GWAS dataset. The details of these seven IVs were presented in Table 1.

**Table 1:** Correlation of instrumental variables with PM_2.5_ and hyperthyroidism.

**SNPs**	**PM**_**2.5**_			**Hypothyroidism**		
	**Beta**	**SE**	***P***	**Beta**	**SE**	***P***
rs114708313	0.024558	0.00447797	4.20E-08	2.81E-03	0.00091972	2.20E-03
rs1372504	0.0122914	0.00221931	3.10E-08	4.32E-05	0.000217876	1.30E-02
rs1537371	0.0123705	0.00214859	8.50E-09	1.48E-05	0.000210865	3.80E-01
rs6749467	−0.0123919	0.00218282	1.40E-08	1.22E-04	0.000214055	9.20E-02
rs12203592	0.113396	0.01913500	3.10E-09	1.17E-03	0.00227423	8.40E-07
rs77205736	0.0135219	0.00241312	2.10E-08	−1.75E-04	0.000236412	2.10E-05
rs77255816	0.0313937	0.00572778	4.20E-08	2.40E-04	0.000563217	2.80E-01

PM_2.5_, particulate matter 2.5; SNP, single-nucleotide polymorphism; Beta, the regression coefficient based on PM_2.5_ raising effect allele; SE, standard error.

### Pleiotropy and heterogeneity analysis

Both MR-Egger intercept and the MR-PRESSO test showed no significant pleiotropy (*P* > 0.05, Table 2). This indicated that the seven SNPs do not affect hypothyroidism through biological pathways independent of PM_2.5_ exposure. In addition, both MR-Egger and IVW showed *P* > 0.05 in Cochran's *Q*-test (Table 2), indicating that the seven genetic variants of PM_2.5_ did not have significant heterogeneity in the hypothyroidism GWAS dataset. Therefore, we could use the seven selected genetic variants of PM_2.5_ exposure as effective IVs for TSMR analysis.

**Table 2:** Pleiotropy and heterogeneity test of PM_2.5_ genetic instrumental variables in GWAS for hypothyroidism.

**Pleiotropy test**				**Heterogeneity test**					
**MR-egger**			**PRESSO**	**MR-egger**			**Inverse variance weighted**		
**Intercept**	**SE**	***P***	***P***	**Q**	**Q_df**	***P***	**Q**	**Q_df**	***P***
−0.0003	0.0009	0.76	0.21	10.02	5	0.07	10.23	6	0.12

GWAS, genome-wide association study; PM_2.5_, particulate matter 2.5; SE, standard error; *P* ≥ 0.05 represents no significant pleiotropy; *P* ≥ 0.05 represents no significant heterogeneity.

### TSMR analysis of PM_2.5_ level and hypothyroidism

**Figure 2:**
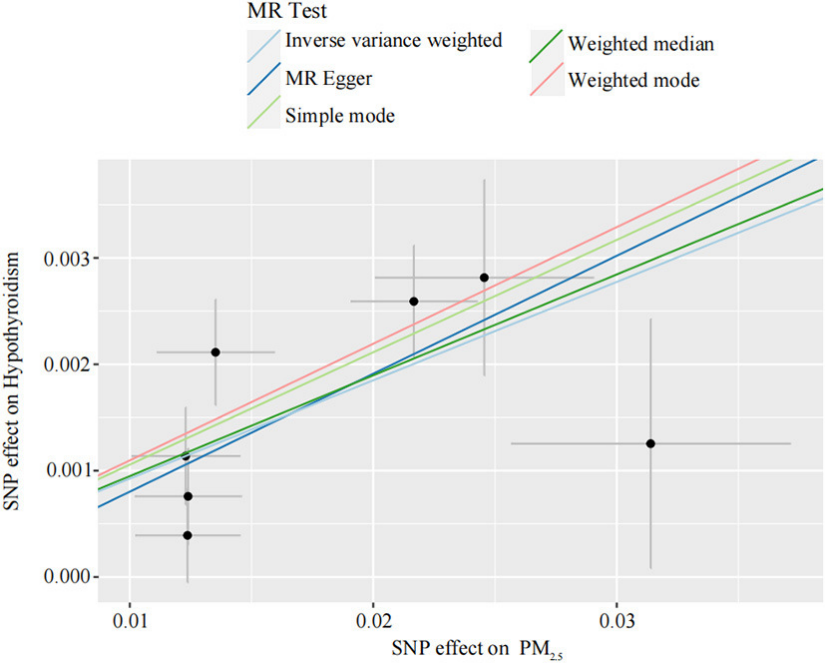
Individual estimates about the putative causal effect of PM_2.5_ on hypothyroidism. The x-axis shows the SNP effect and SE on each PM_2.5_. The y-axis shows the SNP effect and SE on hypothyroidism. The regression line for MR Egger, weighted median, inverse variance weighted, simple mode, and weighted mode is shown. PM_2.5_, particulate matter 2.5; SNP, single nucleotide polymorphism; SE, standard error.

**Figure 3:**
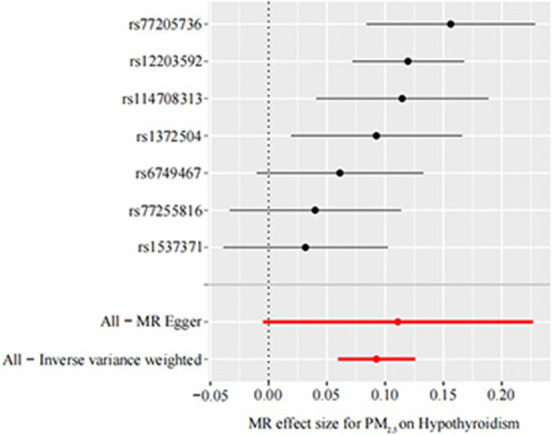
Forest plot of PM_2.5_ associated with risk of hypothyroidism. The x-axis shows the MR effect size for PM_2.5_ on hypothyroidism. The y-axis shows the analysis for each of the SNPs. MR, Mendelian randomization; PM_2.5_, particulate matter 2.5; SNP, single nucleotide polymorphism.

Our TSMR analysis revealed the following odds ratios (ORs) and 95% confidence intervals (CIs): (1) in the MR-egger model, OR = 1.12, 95% CI = 1.00–1.25, *P* = 0.119; (2) in the weighted median model, OR = 1.10, 95% CI = 1.06–1.14, *P* < 0.001; (3) in the IVW model, OR = 1.10, 95% CI 1.06–1.13, *P* < 0.001; (4) in the simple model, OR = 1.11, 95% CI 1.04–1.19, *P* < 0.05; and (5) in the weighted model, OR = 1.12, 95% CI = 1.05–1.19, *P* < 0.05. Although the MR-Egger model results were not significant, the IVW and median weighted model results were significant, and the ORs of the five models are all positive. This finding indicated that each standard error increase in the level of PM_2.5_ exposure was significantly associated with increased risk of hypothyroidism (Figure 2, Table 3). As shown in Figure 3, the regression lines obtained by these five methods were in the same direction, and the promoting effect of a single SNP on hypothyroidism increased as the effect of a single SNP on the PM_2.5_ exposure level increases.

**Table 3:** Two-sample Mendelian randomization analysis results between PM_2.5_ and hypothyroidism.

**Exposure**	**Outcome**	**Method**	**OR**	**95%CI**	***P***
PM_2.5_	Hypothyroidism	MR egger	1.12	(1.00, 1.25)	1.19E-01
		Weighted median	1.10	(1.06, 1.14)	1.95E-06
		Inverse variance weighted	1.10	(1.06, 1.13)	2.93E-08
		Simple mode	1.11	(1.04, 1.19)	2.23E-02
		Weighted mode	1.12	(1.05, 1.19)	1.50E-02

PM_2.5_, particulate matter 2.5; OR, odds ratio; CI, confidence interval; the significance was at *P* < 0.05.

### Sensitivity analysis

**Figure 4:**
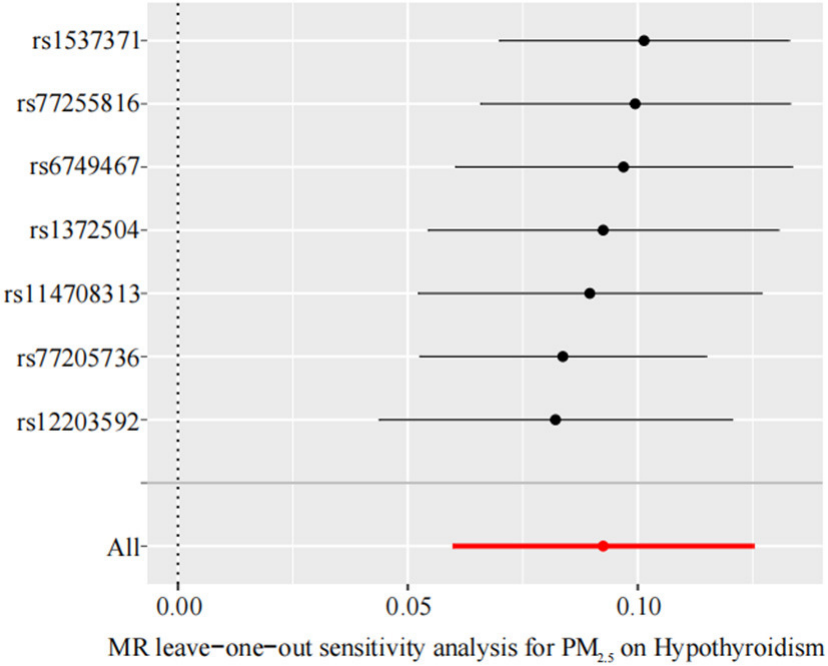
Leave-one-out sensitivity analysis for the effect of PM_2.5_ on hypothyroidism. The x-axis shows MR leave-one-out sensitivity analysis for PM_2.5_ on hypothyroidism. The y-axis shows the analysis for the effect of leave-one-out of SNPs on hypothyroidism. MR, Mendelian randomization; PM_2.5_, particulate matter 2.5; SNP, single nucleotide polymorphism.

We performed sensitivity analysis of the TSMR results by using the leave-one-out method to determine whether the MR results were sensitive to an IV. Each black dot in the forest plot represents a TSMR analysis (using the IVW method), excluding that specific SNP; an overall analysis including all SNPs is shown for comparison (Figure 4). The lines of all IVs are on the right side of 0. Moreover, removing each SNP does not have a fundamental impact on the results. The TSMR results in this study are relatively robust, suggesting that PM_2.5_ is a risk factor for hypothyroidism.

## Discussion

To date, a number of epidemiological studies have found that specific pollutants in the air are risk factors for hypothyroidism (37–40), but due to the methodological limitations of traditional observational studies, it is difficult to determine the causal relationship between the two. MR is based on the premise that human genetic variants are randomly distributed in the population and that these genetic variants are largely independent of confounders, and can be used as IVs to assess the causal association between exposure and outcome (41). In the present study, we assessed the causal association between PM_2.5_ concentrations and the risk of hypothyroidism using TSMR analysis based on a large-scale GWAS dataset. We found that in the European population, increased PM_2.5_ concentrations are associated with an increased risk of hypothyroidism. Our findings indicate a strong causal relationship between PM_2.5_ concentrations and hypothyroidism.

A large epidemiological survey of five cohorts from Europe and the United States found that higher PM_2.5_ concentrations are associated with higher odds of hypothyroidism in pregnant women (OR 1.21 per 5 μg/m^3^ change; 95% CI 1.00–1.47) (42). Other studies have reported that PM_2.5_ exposure affects thyroid function and thyroid hormone secretion. Wang et al. found that PM_2.5_ exposure during pregnancy is significantly negatively correlated with maternal serum FT4 levels (43), and there have been similar results in pregnant women in other regions (44, 45). In traditional observational epidemiological studies, confounding factors often interfere with the results, making the interpretation of etiology unreliable. This study is the first to investigate the causal relationship between PM_2.5_ concentrations and hypothyroidism using TSMR. Our findings are similar to those of traditional observational studies, showing that elevated PM_2.5_ concentrations are significantly associated with an increased risk of hypothyroidism (OR 1.10, 95% CI 1.06–1.13, *P* = 2.93E-08). These findings show that the causal association between genetic variation in PM_2.5_ and increased hypothyroidism risk is robust. Therefore, improving air quality and reducing PM_2.5_ concentrations can effectively reduce the risk of hypothyroidism.

The mechanism by which PM_2.5_ increases the risk of hypothyroidism remains unclear. Compared with PM_10_, PM_2.5_ has a smaller particle size and can reach the distal lung segments including the alveoli, enter the blood, and penetrate the blood barriers of multiple organs such as the brain, liver, and kidney, posing a greater threat to health (46, 47). Studies have found that PM_2.5_ can inhibit the gene expression and activity of endogenous antioxidant enzymes (48), activate the body's oxidative stress response, and promote dysfunction in multiple organs and systems (49, 50). Previous studies have reported that increased traffic-related PM_2.5_ concentrations are associated with altered responses to inflammatory markers (51). Animal experiments have found that artificial PM_2.5_ exposure can induce increased levels of interleukin (IL)-1, IL-6 and tumor necrosis factor α (TNFα) in rats, thereby increasing the risk of nasal lesions (52). Hypothyroidism is associated with disturbed cytokine concentrations, an abundance of reactive oxygen species (ROS), and altered signal transduction in most parts of the brain (53). In addition, chronic inflammation plays an important role in the pathogenesis of many diseases, including hypothyroidism (54). A recent experimental study in female rats found that PM_2.5_ exposure reduces circulating thyroid hormone levels by interrupting thyroid hormone biosynthesis, biotransformation, and transport; by inducing oxidative stress and inflammatory responses; and ultimately by activating the hypothalamic–pituitary–thyroid axis and inducing the production of hepatic transthyretin (55). Epidemiological studies found that a 10 μg/m^3^ increase in PM_2.5_ is associated with a 0.12 μmol/L decrease in FT4 and a 0.07 μmol/L increase in FT3, and the FT4/FT3 ratio is negatively correlated with PM_2.5_ (56). Based on the above findings, we hypothesize that high PM_2.5_ concentrations induce oxidative stress and inflammatory responses in the body, which in turn deregulate thyroid hormone secretion, decrease serum FT4 levels, and increase the incidence of hypothyroidism.

Our MR study has several advantages. First, we used TSMR to analyze the causal relationship between PM_2.5_ concentrations and hypothyroidism, making up for the insufficiency of traditional observational studies and adding new evidence for assessing the health risks of environmental pollutants. Second, this study benefits from large-scale PM_2.5_ GWAS (*n* = 423,796 individuals from Europe) and hypothyroidism GWAS (*n* = 462,933 individuals from Europe) datasets. Moreover, because the individuals are all of European descent, the impact of potential associations caused by population stratification have likely been reduced. Third, we used multiple independent genetic variants as a tool to reduce the impact of linkage disequilibrium on potential associations. Fourth, we selected multiple approaches for MR analysis and performed a comprehensive pleiotropy analysis to assess these. The potential association between genetic variation in PM_2.5_ levels with known risk of hypothyroidism warrants robust results.

This study also has some limitations. First, the TSMR analysis is based on European ancestry, and this relationship may change in individuals of other ancestries. Hence, TSMR analysis should also be performed in individuals of at least one other ancestry. Second, we only used summary statistics for MR analysis, and can only make a preliminary judgment on the causal relationship between PM_2.5_ and hypothyroidism. The specific mechanism of how PM_2.5_ increases the risk of hypothyroidism still needs further research.

## Conclusion

We have provided genetic evidence that high PM_2.5_ concentrations can increase the risk of hypothyroidism. Our findings may have public health implications to raise awareness of the extent to which air quality is associated with the risk of hypothyroidism. This may provide guidance for the prevention and treatment of hypothyroidism.

## Data availability statement

Publicly available datasets were analyzed in this study. This data can be found here: https://gwas.mrcieu.ac.uk/datasets/.

## Ethics statement

This article contains human participants collected by several studies to report the large-scale GWAS for PM_2.5_ and for hypothyroidism. All participants gave informed consent in all the corresponding original studies, as described in the Materials and methods.

## Author contributions

YZ and YueW: designing the study. SL and YueW: carrying out the study, analyzing the data, and writing the article. YunW: revising the article. All authors read and approved the final manuscript.

## Conflict of interest

The authors declare that the research was conducted in the absence of any commercial or financial relationships that could be construed as a potential conflict of interest.

## Publisher's note

All claims expressed in this article are solely those of the authors and do not necessarily represent those of their affiliated organizations, or those of the publisher, the editors and the reviewers. Any product that may be evaluated in this article, or claim that may be made by its manufacturer, is not guaranteed or endorsed by the publisher.
