# Supplementary material for: Causal relationship between particulate matter 2.5 and hypothyroidism: A two-sample Mendelian randomization study
Source: Front Public Health. 2022 Nov 25;10:1000103. doi: 10.3389/fpubh.2022.1000103 (PMC9732245; doi:10.3389/fpubh.2022.1000103)
Supplement: Supplementary file 1 [file Table_1.docx]

| **Supplementary Table 1 Genome-wide association study (GWAS) for hypothyroidism.** | | | | | | | |  |
| --- | --- | --- | --- | --- | --- | --- | --- | --- |
| GWAS ID | Year | Trait | ncase | ncontrol | nsnp | Population | Sex |  |
| ukb-b-19732 | 2018 | hypothyroidism | 22687 | 440246 | 9851867 | European | Men and Women |  |
| *GWAS ID, Genome-wide association study identity; ncase, the number of hypothyroidism case; ncontrol, the number of the control; nsnp, the number of single-nucleotide polymorphism.* | | | | | | | |  |
|  |  |  |  |  |  |  |  |  |
|  |  |  |  |  |  |  |  |  |
