# Supplementary material for: Causal relationship between particulate matter 2.5 and hypothyroidism: A two-sample Mendelian randomization study
Source: Front Public Health. 2022 Nov 25;10:1000103. doi: 10.3389/fpubh.2022.1000103 (PMC9732245; doi:10.3389/fpubh.2022.1000103)
Supplement: Supplementary file 3 [file Table_3.docx]

| **Supplementary Table 3 Eight genetic variants significantly associated with PM_2.5_ exposure as instrumental variables.** | | | | | | |
| --- | --- | --- | --- | --- | --- | --- |
| SNP | Effect allele | Other allele | Effect allele frequency | Beta | SE | *P* |
| rs114708313 | T | A | 0.07 | 0.025 | 0.0045 | 4.20E-08 |
| rs12203592 | T | C | 0.21 | 0.022 | 0.0026 | 6.20E-17 |
| rs1372504 | A | G | 0.37 | 0.012 | 0.0022 | 3.10E-08 |
| rs1537371 | A | C | 0.50 | 0.012 | 0.0021 | 8.50E-09 |
| rs6749467 | A | G | 0.47 | -0.012 | 0.0022 | 1.40E-08 |
| rs72642437 | T | C | 0.004 | 0.113 | 0.0191 | 3.10E-09 |
| rs77205736 | T | C | 0.27 | 0.014 | 0.0024 | 2.10E-08 |
| rs77255816 | T | C | 0.04 | 0.031 | 0.0057 | 4.20E-08 |
| *PM_2.5_, particulate matter 2.5; SNP, single-nucleotide polymorphism；Beta, the regression coefficient based on PM_2.5_ raising effect allele; SE,* *standard error; Eight SNPs with P < 5 × 10^−8^ were selected as independent genetic instrumental variants; linkage disequilibrium region width k = 5000 kb, linkage disequilibrium coefficient r^2^ < 0.01.* | | | | | | |
